# Integrated FRET Polymers Spatially Reveal Micro‐ to Nanostructure and Irregularities in Electrospun Microfibers

**DOI:** 10.1002/advs.202304488

**Published:** 2023-10-28

**Authors:** Xiaojian Liao, Dmitrii Sychev, Khrystyna Rymsha, Mahmoud Al‐Hussein, José Paulo Farinha, Andreas Fery, Quinn A. Besford

**Affiliations:** ^1^ Macromolecular Chemistry Bavarian Polymer Institute University of Bayreuth 95440 Bayreuth Germany; ^2^ Technische Universität Dresden Chair for Physical Chemistry of Polymeric Materials Faculty of Chemistry and Food Science 01069 Dresden Germany; ^3^ Physics Department and Hamdi Mango Center for Scientific Research The University of Jordan Amman 11942 Jordan; ^4^ Centro de Quimica Estrutural Department of Chemical Engineering Instituto Superior Técnico Universidade de Lisboa Lisboa 1049‐001 Portugal; ^5^ Leibniz‐Institut für Polymerforschung Dresden e.V. Hohe Str. 6 01069 Dresden Germany

**Keywords:** conformation reporting, electrospinning, FRET, messenger materials, polymers

## Abstract

A spatial view of macroscopic polymer material properties, in terms of nanostructure and irregularities, can help to better understand engineering processes such as when materials may fail. However, bridging the gap between the molecular‐scale arrangement of polymer chains and the spatially resolved macroscopic properties of a material poses numerous difficulties. Herein, an integrated messenger material that can report on the material micro‐ to nanostructure and its processes is introduced. It is based on polymer chains labeled with fluorescent dyes that feature Förster resonance energy transfer (FRET) dependent on chain conformation and concentration within a host polymer material. These FRET materials are integrated within electrospun polystyrene microfibers, and the FRET is analyzed by confocal laser scanning microscopy (CLSM). Importantly, the use of CLSM allows a spatial view of material nanostructure and irregularities within the microfibers, where changes in FRET are significant when differences in fiber geometries and regularities exist. Furthermore, changes in FRET observed in damaged regions of the fibers indicate changes in polymer conformation and/or concentration as the material changes during compression. The system promises high utility for applications where nano‐to‐macro communication is needed for a better understanding of material processes.

## Graphical abstract

Probing polymer material nanostructure and processes that are spatially resolved poses numerous difficulties. Herein, Förster resonance energy transfer (FRET) polymers are integrated into electrospun fibers so as to inform on material structure and properties in a spatial sense by confocal laser scanning microscopy, which informs readily where material properties are changing.

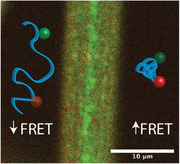

## 1 Introduction

Developing methods for extracting information on the micro‐ to nanoscopic environment within polymeric materials poses numerous challenges, particularly when the area of interest is away from physically accessible surfaces. Often this comes down to an inability to spatially localize structure and features, which can include component inhomogeneities,^[^ ^1^ ^]^ regions of crystallinity,^[^ ^2^ ^]^ and rearrangements due to dynamic force transmission.^[^ ^3^ ^]^ The benefit of resolving these features is clear, where it can allow for a better understanding of the properties of a material, both in biological^[^ ^4^ ^]^ and engineering contexts,^[^ ^5^ ^]^ such as imperfections, stress, and damage. Methods for spatially probing material properties can vary widely, depending on the material type, but can include magneto‐optical imaging,^[^ ^6^ ^]^ pump‐probe microscopy methods,^[^ ^7^ ^]^ fluorescence X‐ray,^[^ ^8^ ^]^ computed tomography scanning,^[^ ^9^ ^]^ reflectance confocal microscopy,^[^ ^10^ ^]^ and Raman microscopy.^[^ ^11^ ^]^ However, these tend to be specific to certain materials, architectures, and the property of interest, which reduces the more broad‐scale use in the physico‐chemical sciences. In this regard, a general spatial reporting system for structure and properties in soft polymeric materials may have a high impact in several fields.

Materials that produce macroscopic signals based on nanoscopic changes in environmental conditions can be leveraged in such applications. This includes self‐reporting polymers that produce signals in response to force and/or stimuli,^[^ ^12^ ^]^ and polymers that are capable of nano‐to‐macro communication of subtle and dynamic/reversible changes in condition (termed messenger materials).^[^ ^13^ ^]^ Such systems can include fluorescence dyes coupled into specific polymer architectures so that the matrix‐dependent conformation and concentration of the reporting chains affect the macroscopic fluorescence signal. This concept could be very useful for probing material properties within bulk materials, which can otherwise remain somewhat hidden.

A target engineering application for probing nanostructure is in electrospun polymer microfibres. It is known that the properties of microfibers (e.g., Young's modulus) can be tailored by managing the assembly of polymer chains at the nanoscale, yet their internal nanostructure is still poorly understood.^[^ ^14^ ^]^ During the electrospinning process, a viscoelastic polymer solution, or melt, elongates into a collinear jet carrying an electric charge, which moves away from the output needle under the effect of an externally applied electrostatic field, before the final fiber reaches the collecting surface.^[^ ^15^ ^]^ The behavior of the polymer chains within the jet can determine the molecular packing and orientation in the final fibers.^[^ ^16^ ^]^ It is known that high‐strength fibers are the result of high macromolecular chain alignment coupled with high crystallinity.^[^ ^17^ ^]^ Therefore, electrospun fibers provide a key material where it is highly desirable to readily identify changes in the micro‐ to nanostructure, spatially, within the fibers, during the manufacturing process.

**Figure 1:**
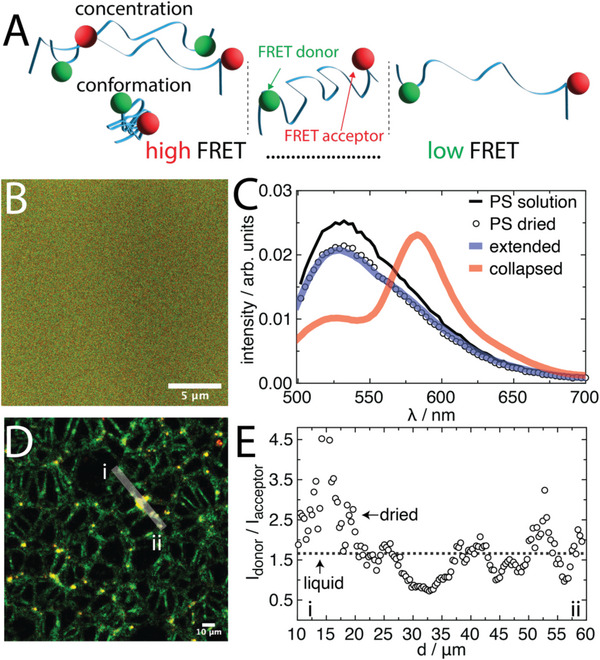
A) Schematic describing the changes in FRET from the messenger material depending on conformation and concentration, B) along with a comparison of the FRET in PS solution, C) with spectra of a wet and dried PS sample, with the FRET polymer in the extended (blue) and D) collapsed form, accompanied with a CLSM image of a dried PS/FRET sample (on the air interface side), E) along with line profile analysis. The scale bars are 5 µm (B) and 10 µm (D).

Herein, we approached this problem by integrating a very small concentration of Förster resonance energy transfer (FRET)‐integrated messenger polymers into polystyrene (PS) solutions that were used for electrospinning microfibers. The FRET polymers were designed so that the efficiency of FRET reflects polymer conformation and/or concentration in the fibers (**Figure** **1**A), which could be readily spatially resolved with confocal laser scanning microscopy (CLSM). This allowed for identification of the micro‐ to nanostructure within the fibers. Regions that contained natural imperfections due to the electrospinning process, as well as those that were physically damaged by micromanipulation, could be spatially resolved, with the integrated FRET polymers not changing the native PS microfiber structure. Our methods, which are not restricted to the present PS system, can offer a new basis for the general integration of messenger materials for reporting micro‐ to nanostructure and irregularities in polymer materials.

## 2 Results and Discussion

FRET is a process by which a fluorophore (the donor) in an excited state transfers its energy to a neighboring molecule (the acceptor) through a non‐radiative dipole‐dipole interaction, which is strongly distance‐dependent between the donor and acceptor molecules.^[^ ^18^ ^]^ The FRET process can therefore be used to understand the distance between the donor and acceptor molecules. We developed a FRET‐integrated diblock random copolymer consisting of a poly(*N*‐isopropropylacrylamide) (PNIPAM) backbone, with a nitrobenzofurazan‐based FRET donor in one block, and a rhodamine B‐based acceptor in the other block.^[^ ^19^ ^]^ This system has been shown previously to produce FRET that is strongly dependent on the conformation of the individual chains that change the distance between the donor and acceptor molecules,^[^ ^19^ ^]^ which is our chosen messenger material to explore for integration into a second medium. We chose PS as a material mixture with which to produce well‐defined microscopic fibers by electrospinning. The key requirement for integrating a messenger polymer into a different material matrix is complete miscibility between the two polymers, which depends on complex interactions toward each polymer, and toward themselves.^[^ ^20^ ^]^ If the polymers are immiscible, then the reported messages would reflect the miscibility rather than some inherent material feature.

When the FRET polymers were mixed into PS solutions in DMF, CLSM showed a homogenous mixture (Figure 1B), and the fluorescence spectrum (Figure 1C) indicated that the FRET‐pairing in the mixture was consistent with an extended chain conformation (i.e., high donor‐to‐acceptor ratio), also for the dry‐state sample. The extended chain conformation in Figure 1C (from the FRET polymer in ethanol), shows mostly the emission of the donor dye, whilst the collapsed conformation (from the FRET polymer in a 30% v/v mixture of ethanol and water, a co‐nonsolvency mixture that causes chain collapse) shows the decrease in the emission of the donor dye and consequent appearance of emission from the acceptor dye.^[^ ^19^ ^]^ Interestingly, when the mixture was allowed to dry on a quartz surface (Figure 1D), and the CLSM imaging was performed on the air‐interface side, the donor intensity (green) was found to significantly increase in some regions, while also small regions of high acceptor intensity (red) were seen in the composite images. This is likely due to different chain relaxation dynamics and packing while the solvent was evaporated within the PS host material. Line profile analysis (Figure 1E) confirmed that the FRET between the dry and wet states were somewhat similar, but there were clear deviations in the dry state in comparing across regions of high acceptor intensity. This perhaps reflects some segregation of the reported polymer chains amongst the PS chains, or native imperfections amongst the PS chains, as the system dries, with high FRET efficiency regions only present in spherical regions of ≈1 µm in diameter.

**Figure 2:**
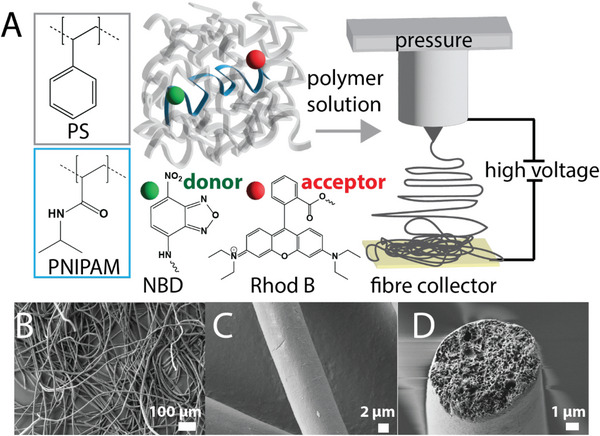
Schematic for the A) incorporation of the FRET polymers into electrospun PS fibers, along with B–D) SEM images of the resulting fibers. The scale bars indicate B) 100 µm, C) 2 µm, and D) 1 µm. PS, poly(styrene); PNIPAM, poly(*N‐*isopropylacrylamide); NBD, nitrobenzofurazan; Rhod B, rhodamine B.

With the miscibility of the two polymers validated on average for a set concentration over the bulk solution (Figure 1C), this mixture was then used as a feedstock for electrospinning fibers (**Figure** **2**A), as well as the same PS solution without the FRET polymer. The applied voltage and flow rate were chosen to aim for fibers of ≈10 µm in diameter, allowing for straight‐forward diffraction‐limited analysis by CLSM later on. The resulting fibers showed good surface structure (Figure 2B,C) as well as a porous inner center (Figure 2D), consistent with previous work on PS electrospun fibers.^[^ ^21^ ^]^ The average diameter of the FRET‐integrated fibers was 9.4 ± 1.2 µm (Figures S1 and S2, Supporting Information), which compared well to the fibers without FRET polymers (8.6 ± 0.94 µm).

An important aspect to validate for this system is that the integration of the FRET polymers did not changed the native structure of the PS microfibers (i.e., the system has not changed the message). Wide‐angle X‐ray scattering (WAXS) and small‐angle X‐ray scattering (SAXS) measurements were performed on the electrospun fibers in order to investigate the effect of incorporating the FRET polymer on the internal structure of the FRET/PS electrospun fibers from the Angstrom to nanometer length scale, respectively. Two isotropic broad halos at scattering vectors *q*_1_ = 7.40 and *q*_2_ = 13.73 nm^−1^ were observed in the WAXS pattern of the neat PS fibers indicating the highly amorphous nature of the PS fibers (Figure S3E, Supporting Information). The isotropic intensity distribution of both halos indicated that the PS chains were essentially randomly distributed within the electrospun fibers. The PS/FRET fibers exhibited very similar 2D WAXS patterns to those of the PS fibers. The two isotropic halos were slightly shifted to higher *q* values: *q*_1_ = 7.47 and *q*_2_ = 13.83 nm^−1^, respectively. However, both halos remained isotropic with no preferred chain orientation. Meanwhile, the 2D SAXS patterns of both PS and PS‐FRET fibers exhibited only isotropic scattering around the beam‐stop because the fibers were randomly distributed, with no discernible correlation peaks (Figure S3F, Supporting Information). This confirmed the absence of any internal electronic contrast from correlated crystalline domains or lamellar nanostructures within both fibers in the investigated *q* range as a result of their amorphous nature. It is possible that the integrated FRET polymers are at too low a concentration to be seen by the SAXS/WAXS measurements, however, we infer that the incorporation of the FRET reporting polymer in the PS electrospun fibers did not induce any significant changes in the packing of the PS chains and in turn had no noticeable effect on the internal Angstrom to nanostructure length scale of the PS fibers. This may be different for other polymer fiber systems that exhibit crystallinity.

**Figure 3:**
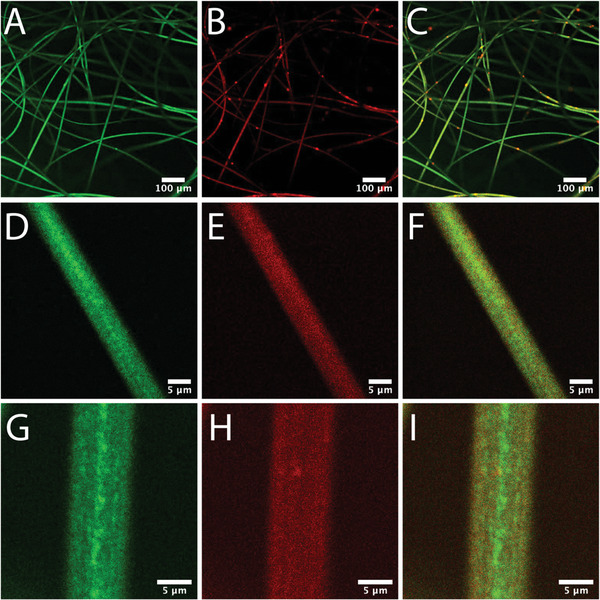
CLSM analysis of the PS‐FRET fibers on a glass cover slide across the donor (green, A,D,G), acceptor (red, B,E,H), and composite (C,F,I) channels. Excitation was 458 nm, with the donor channel collected between 482–547 nm and the acceptor between 568–700 nm. The scale bars indicate (A–C) 100 µm and (D–I) 5 µm.

The fibers were then examined by CLSM with the channels split into donor (green) and acceptor (red), along with a merged composite (**Figure** **3**). Interestingly, in the resulting fibers, occasional regions of increased acceptor intensity were found (Figure 3B,C), which is consistent with what was found for the dried PS‐FRET solution in Figure 1D, likely indicating regions of differing polymer density and/or conformation. More importantly, the resulting images across different length scales demonstrated that in the center of the fibers, there was a region of increased donor intensity (Figure 3A,D,F), not seen in the acceptor channel (Figure 3B,E,H), which was most clear in the merged channel (Figure 3C,F,I). This spine‐like region was found to persist along most fibers in a consistent way, visible at least over 50 µm before the fibers bend out of the focal plane. Importantly, the neat PS fibers did not exhibit any significant fluorescence or scattering effects (almost 10‐fold less) in comparison to the PS‐FRET system (Figure S4, Supporting Information), confirming that the origin of the signals observed in Figure 3 was the result of the FRET polymer and not scattering or autofluorescence effects from the PS.

The fibers were further examined by Raman microscopy, where specific bands corresponding to either PS or FRET polymers were selected (Figure S5, Supporting Information). The resulting spatially‐resolved images showed no spine‐like features along the PS‐FRET fibers at the two laser wavelengths selected (532 and 785 nm, Figures S6 and S7, Supporting Information, respectively). Recently, Areias et al. explored the use of reflectance confocal microscopy (RCM) as a tool for characterizing the structural morphology of colloidal crystals.^[^ ^10^ ^]^ The RCM method is based on measuring the backscattered light from a sample, where contrast is produced by refractive index differences within media due to long‐range order. We used this approach to analyze the neat PS fibers. Interestingly, spine‐like structures identical to those exhibited by the FRET polymer were observed at the longer RCM wavelength range (Figure S8, Supporting Information). It is not clear at this stage why the longer wavelength RCM revealed the spine‐like structures. Nonetheless, it demonstrates that CLSM of the FRET systems can spatially resolve internal structure and features of the electrospun PS fibers. Moreover, when a PS‐FRET fiber end was analyzed, no localized brightness was observed (Figure S9, Supporting Information), ruling out the possibility that wave‐guiding of the fluorescence signal was the cause of the spine‐like structures.

**Figure 4:**
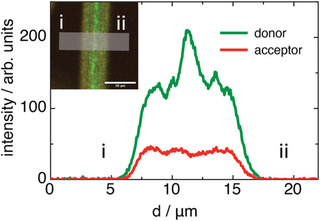
Line profile analysis across donor and acceptor channels for a FRET‐integrated microfiber (shown in insert). Light grey box indicates the approximate region over which the line profile was calculated. The scale bar indicates 10 µm.

The signals observed for the PS‐FRET fibers were further analyzed by line‐profile analysis (**Figure** **4**) across the fibers. It was readily apparent that the peak in the donor intensity dwarfed any signal from the acceptor, where the acceptor had a reduction in intensity in the center of the fiber, consistent with a decrease in FRET as the chains exhibited a more stretched conformation, possibly due to chain alignment in the core, or to a region where all chains exhibit stretched conformation in all directions. The width of the spine‐like region was ≈30% of the width of the fiber itself, which is consistent with what has been observed for internal nanostructure on other fiber types. For instance, cross‐sectional mapping of Young's modulus of poly[2‐methoxy‐5‐(2‐ethylhexl‐oxy)−1,4‐phenylene‐vinylene] polymer microfibers revealed a region of ≈30% of the width of the fibers had an increased stiffness due to a denser polymer core.^[^ ^14^ ^]^ This is for a drastically different fiber system, but the consistency is interesting.

The region we have observed in the PS‐FRET fibers is most likely due to changes in the conformation of the polymer chains toward the core of the microfibers, but the PS is amorphous, with no crystallinity or strong orientations revealed by WAXS (Figure S3, Supporting Information), so the nature of the conformational changes are not clear. It is not possible to rule out solvent entrapment effects within pores that change the FRET polymer conformation, however, given that the regions of the spine‐like structures extend over 2.5 µm across the fiber diameter, it is not likely that this is due to pores in the structure, which are of the order of 100s of nm. To determine if the increased donor signal at the fiber core was due to low FRET, or some other effect, a photobleaching experiment was performed, whereby the acceptor was bleached with a 546 nm laser. It was seen that after bleaching the acceptor for an extended period of time, there was a small increase in donor intensity (Figure S10, Supporting Information), confirming a degree of native FRET within the material, which is consistent with the spine‐like structures resulting from changes in chain conformation in the middle of the fibers. The integrated system therefore allows direct visualization of the areas where the micro‐ to nanostructure was different.

**Figure 5:**
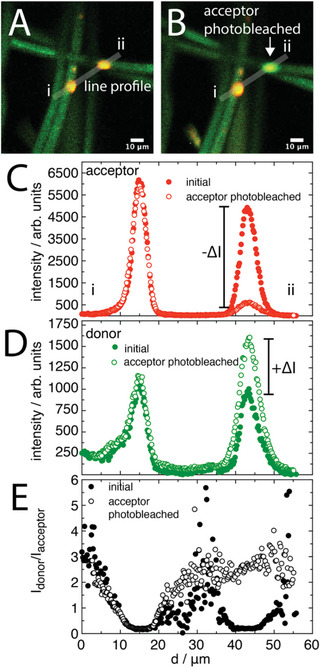
CLSM images of A) integrated FRET fibers that have two clear spots of irregularities, along with the same region after one of the irregularities was subjected to localized photobleaching on the acceptor (over 1.5 h with 100% intensity 546 nm excitation). Line profile analysis was performed across the C) acceptor and D) donor channels, E) along with a FRET ratio. The light grey lines indicate regions over which the line profiles were calculated. Scale bars indicate 10 µm.

The areas of high acceptor intensity (in Figure 3B) were investigated to determine the contribution from FRET. A region on the fibers was found where there were two spots of high acceptor intensity (**Figure** **5**A). One of these regions was then selectively bleached with full laser intensity at the acceptor excitation wavelength (546 nm) for a period over 1.5 h. Subsequently, the bleached spot revealed more intense donor emission (green color in composite image) (Figure 5B). Line profile analysis across the donor and acceptor channels showed that there was minimal change on the spot that was not bleached. However, the bleached spot exhibited a ≈90% reduction in acceptor intensity (Figure 5C), whereas on the donor channel, this same spot increased in intensity by ≈160% (Figure 5D). By taking the intensities over both channels the FRET ratio was calculated (Figure 5E), clearly showing that FRET occurring before bleaching of the acceptor, but this was strongly decreased resulting in I_donor_/I_acceptor_ > 1. However, the donor intensity was still significantly greater than the surrounding fiber area. This indicated that the spot regions of increased acceptor emission are due to increases in polymer density, which we anticipate is due to both components (PS and FRET polymer). This may also include changes in material conformation in these regions, but it is not possible to differentiate the effects of high concentration and changed chain conformations on FRET, as both effects lead to changes in the distance between donors and acceptors (Figure 1A).

**Figure 6:**
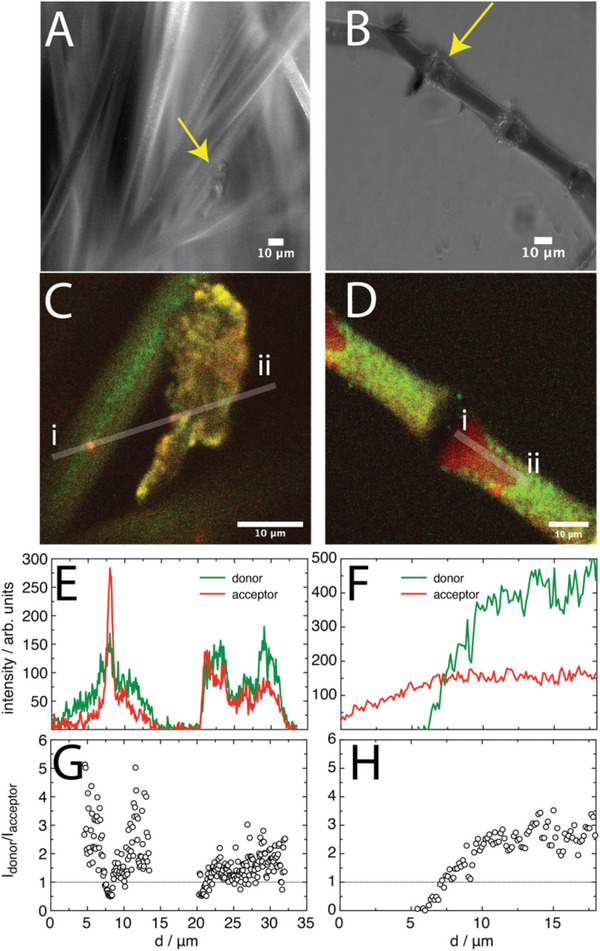
Wide‐field images of A) an irregular fiber, and B) a physically damaged fiber, done so with a micromanipulator device, C,D) along with CLSM images the same (respectively), E,F) with line profile analyses (respectively), and G,H) corresponding FRET ratios (respectively). The yellow arrows in A and B indicate the regions of interest, and the light grey lines in C and D indicate the approximate regions over which the line profiles were calculated. Scale bars indicate 10 µm.

Lastly, CLSM was used to survey regions of material irregularities across the fibers. It was found that whenever irregularities in the PS fiber existed (e.g., non‐cylindrical fiber or oddly shaped end), as identified by wide‐field microscopy (**Figure** **6**A), the intensity of the acceptor channel was increased (Figure 6C), leading to more yellow color in the composite images, compared to the higher donor intensity in all regular fiber regions. Furthermore, when a fiber was damaged on purpose, via the use of a micromanipulator device, it was found that the acceptor intensity increased significantly, accompanied by an almost total reduction in donor intensity (Figure 6B–D). It was observed that this increase in acceptor intensity occurred before fiber breakage (dinted fiber, Figure S11, Supporting Information), however, the fibers were too rigid to do this in a controlled force manner for quantitative comparisons (via colloidal probe atomic force microscopy, for example). When line profile analysis was performed on both systems, along with FRET ratios (Figure 6E–H), it was found that for material irregularities, the FRET ratio reduces to ≈1, whereas in a normal fiber, the FRET ratio was typically 2.5–3.5 away from the spine‐like regions. Interestingly, when the fiber was physically damaged, the donor emission changed significantly, where the surface appeared “blotchy” with higher intensity output than the normal non‐damaged fibers. As the fiber breaks, it is anticipated that the surface structure will relax as the strain is eased, which is perhaps what the donor channel indicates in this case with the “blotchy” appearance. The increase in donor intensity outside of the cut region might result from material compression leading to a greater number of fluorophores on the surface space, however, in the region of the direct cut, this compression may cross the threshold of material density that then leads to enhanced FRET, revealed through the acceptor channel. It is also possible that atmospheric water plays a role in solvating the damaged surface, interacting with the FRET polymer, though this cannot be determined at this point. A comparison of the FRET across native, dinted, and broken fibers (Figure S12, Supporting Information) showed FRET ratios decreasing in accordance with the magnitude of the observable damage, particularly noticeable across the middle spine‐like regions, where the FRET reduced from ≈8 to ≈2.5 for the dinted fiber, and further to ≈1 for the broken fiber (calculated just away from the break). However, since the fibers were too rigid to damage with a carefully controlled force, a greater quantitative comparison of FRET with damage will need to be investigated with a softer material system. The trend with damage suggests that our system can reveal such incremental changes with applied force, which is the subject of a separate study.

Together, our methods allow for the integration of very small quantities of FRET messenger materials within host polymer materials (160 µg FRET polymer into 2 g PS), to report the host material micro‐ to nanostructure and irregularities. Our methods have the potential to be integrated into material production systems, whereby material internal structure and irregularities can be probed by fluorescence spectroscopy integrated into automated lines of production. We have found that, for our reporter system, a FRET ratio ≈1 can be taken as an indicator for the presence of irregularities or damage in the material, providing a simple threshold for understanding changes in material processes. In addition, the FRET messenger material offers the potential to reveal stresses in the host materials, which is typically a challenge for most experimental methods, and the FRET messenger is stable over long periods of time within the host material (Figure S13, Supporting Information). It is worth mentioning that one main limitation of our methods is that the FRET messenger materials must be pre‐designed for each host material. In the case of polymers, the incorporated FRET polymer may demix from the host polymer, leading to false positive results. In this study, the PNIPAM‐based FRET polymer was incorporated in PS at low concentrations with full miscibility, but this will not hold for every system. For our specific system, the solubility of the PNIPAM‐based FRET polymer is complex, as the polymer is a diblock random copolymer, with both end groups still attached. It is known that the phase behavior of PNIPAM changes depending on the copolymer composition and the presence of end‐groups,^[^ ^22^ ^]^ meaning interpretation of solubility is difficult. However, each and every polymer system should be validated for solubility, as in Figure 1, before attempting integration studies. Solubility challenges, where they exist, can be overcome through judicious choice and validation of each polymer system. Therefore, our work lays the foundation for a non‐invasive method for probing spatially resolved internal structures of bulk materials.

## 3 Conclusion

We have developed FRET polymers that provide optical output reflecting the micro‐ to nanostructure and irregularities within a secondary host polymer material, where the FRET polymer is a very minor component of the polymer matrix. We have integrated these FRET polymers at low concentrations in order to probe structure and density within electrospun PS fibers. Our target in this work was electrospun fibers, but this is not our limitation. We have shown how the FRET polymers can reveal details on micro‐ to nanostructure, imperfections, and damage, which is not readily apparent by most other methods. This opens up many questions regarding the properties of the materials we have probed, but in this work, our vision is exactly about the opportunities and questions that arise from these unique polymers. We anticipate significant work going forward to better understand the messages that these FRET polymers send out to us macroscopically.

## 4 Experimental Section

### Materials

All chemicals were of analytical grade and used as received without purification, with the exception of 2‐hydroxyethyl methacrylate (HEMA) and *N*‐isopropylacrylamide (NIPAM) which were purified by passing through an aluminum oxide column prior to reaction, and by recrystallization from hexane (× 2), respectively, to remove inhibitors. High‐purity water (Milli‐Q water) with a resistivity of >18.2 MΩ cm was obtained from an inline Millipore RiOs/Origin water purification system (Millipore Corporation, Massachusetts, USA). Polished single‐crystal (100)‐silicon wafers were obtained from Silicon Materials, Kaufering, Germany, with a native SiO_2_ layer thickness of ≈1.7 nm. Optical fused quartz square coverslips (22 × 22 × 0.2 mm) were obtained from Micro to Nano (Haarlem, The Netherlands). PGMA (*M*_n_ = 15 000 g mol^−1^, *Ð* = 1.6) was obtained from Polymer Source Inc. (Montreal, Canada). 4‐Chloro‐7‐nitrobenzofurazan (NBD‐Cl), ethanolamine, acetonitrile (ACN), acryloyl chloride, thionyl chloride, dichloroethane (DCE), rhodamine B (Rhod B), HEMA, dichloromethane (DCM), NIPAM, azobisisobutynitrile (AIBN), 1,4‐dioxane (anhydrous), tetrahydrofuran (THF), diethyl ether, hexane, methanol, ethanol, and 1‐propanol were obtained from Sigma‐Aldrich. THF was obtained from Acros Organics. Chloroform was obtained from Fisher Chemicals. CDCl_3_ and DMSO‐*d*_6_ were obtained from Eurisotop (Saint‐Aubin, France). Atactic polystyrene (PS) 165 H was obtained from BASF (Ludwigshafen, Germany). The FRET polymers were synthesized exactly as previously reported.^[^ ^19^ ^]^

### Electrospinning

The electrospinning solutions were prepared by dissolving PS (2 g) in DMF (3.94 mL) overnight with stirring at 400 rpm. Then, 160 µL of fresh FRET/DMF diluted solution with a concentration of 1 mg mL^−1^ FRET polymer was added to the PS solutions. After stirring at a speed of 500 rpm for half an hour, the solution was filled into a 5 mL syringe. The syringe was capped with a metal needle (diameter 0.9 mm) and connected to a high‐voltage DC power supply. During the electrospinning process, a stable flow rate of 2.4 mL h^−1^ was controlled by the syringe pump in the electrospinning machine, and a voltage of +12 kV was used for electrospinning. The distance between the metal needle and the rotating collector covered with aluminum foil was ≈25 cm. After the high voltage was applied, the polymer fiber was collected on the collector at a speed of 100 rpm and a diameter of ≈10 cm. The whole electrospinning process was carried out at room temperature and at a humidity of ≈40%. A quartz glass plate was placed on top of the aluminum foil to collect the fiber for 1 min for subsequent CLSM. The polymer fiber membrane on the aluminum was obtained after electrospinning for 1 h. Finally, the PS/FRET fibers were obtained after drying in a vacuum oven at RT for 48 h. Meanwhile, the pure PS fiber without FRET was also prepared under identical conditions. The fibers were stored in plastic containers wrapped in foil, protected from the light, at 23 °C. A stability study was performed by CLSM imaging 10 months after the electrospinning process. For bulk PS/FRET samples (i.e., no fibers), 100 µL of the electrospinning solution was cast into a 24‐well plate reader plate, spread thin on the bottom with a pipette tip, and dried under a stream of argon. The sample was subsequently placed in a vacuum oven at 23 °C for 2 h before measuring fluorescence spectra and taking CLSM images.

### Fluorescence Spectroscopy

All measurements were performed with a multimode microplate reader (Tecan Spark 10 M, Switzerland). Fluorescence measurements of the FRET polymer solution were performed in 96‐well black plates (Molecular Probes, Invitrogen) at a concentration of 25 µg mL^−1^. Fluorescence spectra were recorded (*λ*_exc_ = 454 nm, *dλ* = 2 nm, 495–700 nm) whilst the temperature was maintained at 24 °C.

### SEM

SEM measurements were performed with a FIB‐SEM Neon 40Esb microscope (Carl Zeiss Microscopy GmbH, Germany), equipped with a field emission gun. The fibers were mounted on double‐sided carbon tape for measurements, where fibers were removed that were clearly “sticking up”, which might create cross‐sections. The samples were uncoated and imaged with EHT (accelerating voltage) of 1 kV, and detected with SE2 (Everhart‐Thornley) and InLens detectors.

### SAXS/WAXS

SAXS and WAXS experiments were performed with a GANESHA 300XL+ system (SAXSLAB ApS, Lyngby/Denmark). The instrument was equipped with a Pilatus 300 K detector, a Cu X‐ray source operated at 50 kV/0.6 mA (*λ* = 1.5408 Å), and a three‐slit collimation system. The detector was moved in a vacuum chamber to a sample‐to‐detector distance of 102 and 1052 mm for WAXS and SAXS measurements, respectively. The scattering patterns were recorded in transmission for 2 h at room temperature in vacuum conditions. The 2D SAXS and 2D WAXS patterns were azimuthally integrated to get the corresponding intensity profiles as a function of the scattering vector, $q=\frac{4\pi }{\lambda }\sin\theta$, where 2θ was the scattering angle.^[^ ^23^ ^]^ The background was subtracted after normalizing the total scattering profile by the transmitted intensity.

### Confocal Laser Scanning Microscopy

A combination setup of an Axio Observer Z.1 inverted microscope with an LSM710 confocal laser scanning module (Carl Zeiss Microscopy, Germany) was used. Measurements were performed with either a 10× or 50× in‐air objective. The donor was excited with an argon laser (458 nm) and the acceptor (for non‐FRET measurements) with a helium‐neon laser (543 nm). Unless otherwise stated, the pin‐hole was set at 1.2 µm, with a laser intensity of 1 to 3% and a gain of 850. Note that comparative images (acceptor to donor channel) always have identical acquisition conditions. The donor collection window was 482–540 nm, and the acceptor was 578–703 nm. A dark (no‐collection) window was kept at 38 nm so as to avoid crosstalk between channels as much as possible. For bleaching experiments, the region of interest was subjected to 100% laser intensity at 543 nm (acceptor excitation) so as to bleach the Rhod B with time. Unless otherwise stated, the conditions were maintained at 21 °C and ≈50% RH. For line profile analyses, all images were corrected for background scattering from the cover glass surfaces, unless otherwise stated.

### Damaging Fibres

The fibers were damaged by using the back side of an AFM cantilever chip (NSC 35, Mikromasch Europe, Wetzlar, Germany) as a blade. The chip was mounted on a micromanipulator, positioned over the fiber surface, and pushed until the fiber was cut in half. The fiber was then mounted directly on the confocal microscope and the position was found by wide‐field microscopy, before performing CLSM. We point out that colloidal probe AFM was initially used to damage the fibers, however, the fibers were too rigid to be damaged this way, therefore a micromanipulator device was used.

### Raman Microscopy

Measurements were performed with a Raman Confocal Imaging System WITEC alpha 300R (WITec GmbH, Ulm, Germany). Single Raman measurements were performed with 532 and 785 nm lasers, with a power of 10 mW, a ×20 objective, with an integration time of 0.5 s and 200 accumulations. Raman mapping measurements were performed with 532 nm (10 mW) and 785 nm (30 mW) lasers, a ×100 objective, with an integration time of 0.5 s and 1 accumulation. A measuring point distance of 500 nm was used, with an area of 40 × 40 µm.

### Statistical Analysis

For the fiber dimension counting, a total of 75 microfibers for both systems were individually assessed for diameters from SEM images. These were then averaged for Figure S1 (Supporting Information).

## Conflict of Interest

The authors declare no conflict of interest.

## Supporting information

Supporting Information

## Data Availability

The data that support the findings of this study are available from the corresponding author upon reasonable request.
