# Supplementary material for: Integrated FRET Polymers Spatially Reveal Micro‐ to Nanostructure and Irregularities in Electrospun Microfibers
Source: Adv Sci (Weinh). 2023 Oct 28;10(36):2304488. doi: 10.1002/advs.202304488 (PMC10754101; doi:10.1002/advs.202304488)
Supplement: Supplementary file 1 — Supporting Information [file ADVS-10-2304488-s001.pdf]

ADVANCED SCIENCE
Open Access

Supporting Information

for *Adv. Sci.*, DOI 10.1002/advs.202304488

Integrated FRET Polymers Spatially Reveal Micro- to Nanostructure and Irregularities in Electrospun Microfibers

*Xiaojian Liao, Dmitrii Sychev, Khrystyna Rymsha, Mahmoud Al-Hussein, José Paulo Farinha, Andreas Fery and Quinn A. Besford**

Supporting Information

Integrated FRET Polymers Spatially Reveal Micro- to Nanostructure and Irregularities in Electrospun Microfibers

*Xiaojian Liao, Dmitrii Sychev, Khrystyna Rymsha, Mahmoud Al-Hussein, José Paulo Farinha, Andreas Fery, Quinn A. Besford**

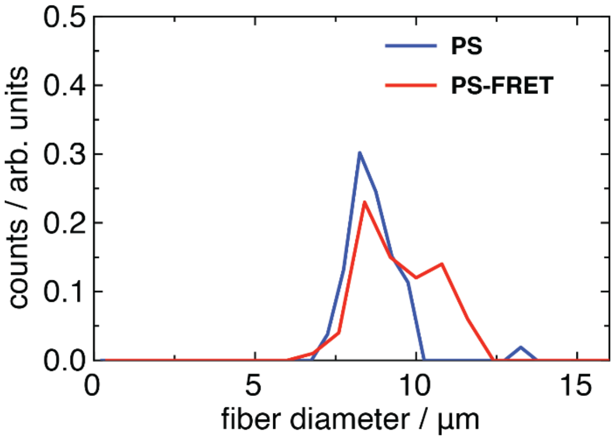

Figure S1. Histogram of counts of fiber diameters for the PS and PS-FRET systems, as determined from multiple SEM images. In total 75 fibers were measured.

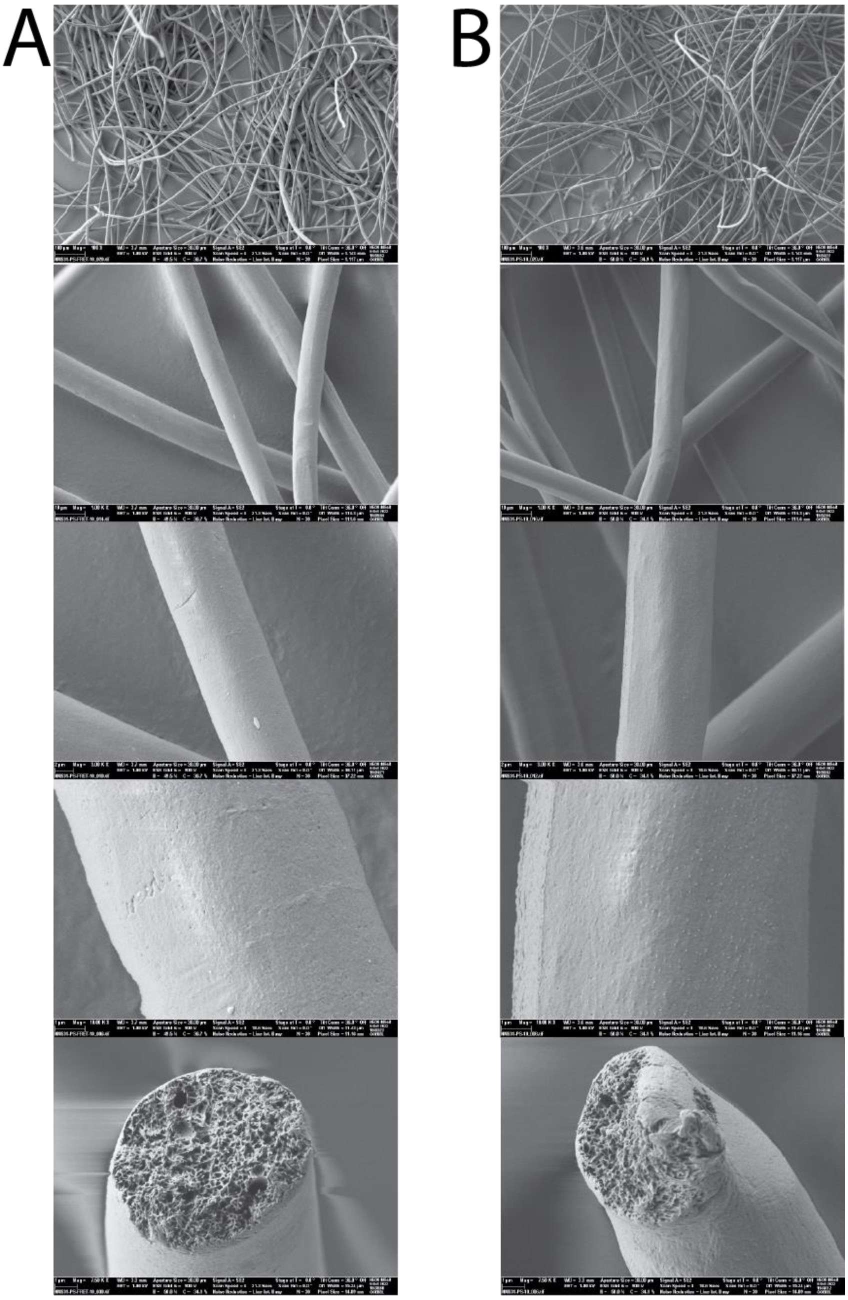

Figure S2. SEM images of different magnifications of the electrospun fibers (A) with and (B) without the integrated FRET polymers. Note some charging artifacts (wavey composition) due to the In-Lens detector at higher magnifications. Note individual scale bars for each image.

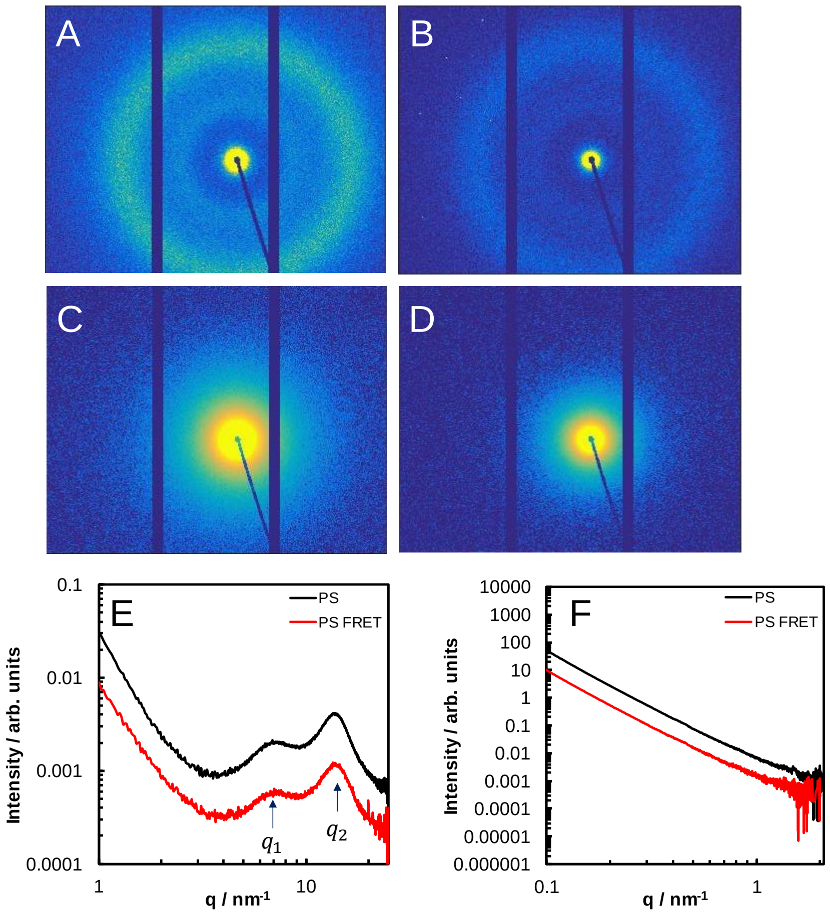

Figure S3. A) 2D WAXS patterns of electrospun PS fibers, and B) PS/FRET fibers. C) 2D SAXS patterns of electrospun PS fibers, and D) PS/FRET fibers. E) Radial integration WAXS profiles, and F) SAXS profiles.

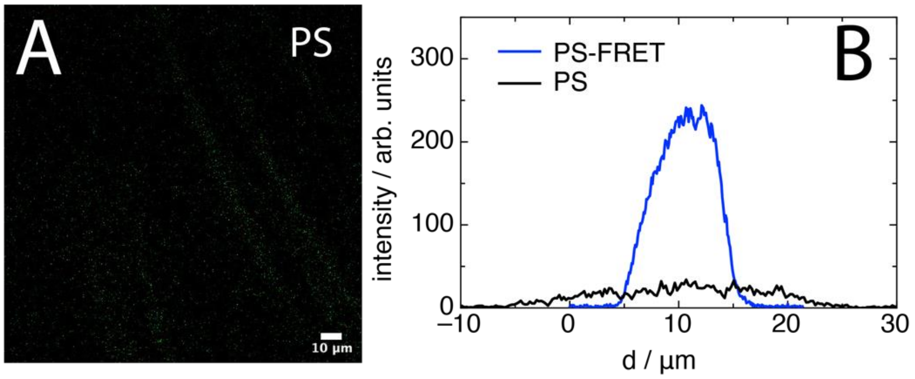

Figure S4. A) CLSM image of PS microfibers under the exact same conditions as for the PS-FRET systems of Figure 3 of the main text, along with B) an intensity line profile of a PS microfiber in comparison to a PS-FRET microfiber. Scale bar indicates 10 µm.

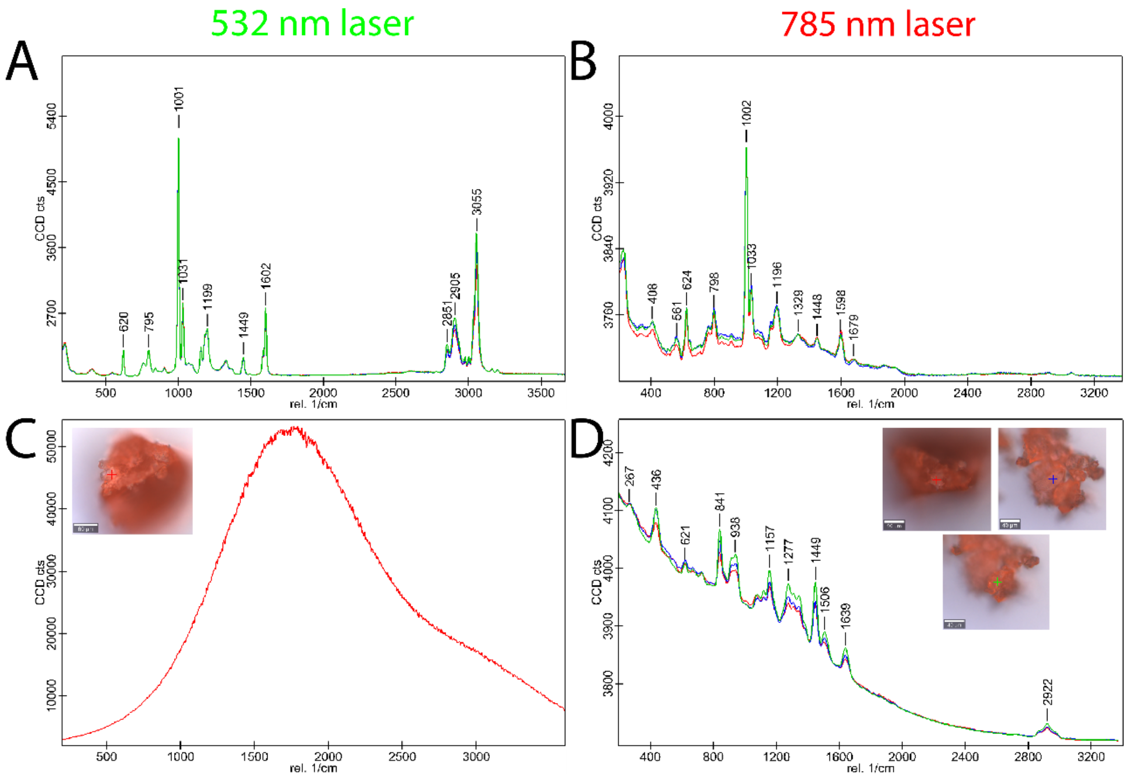

Figure S5. Raman spectra of the PS fibers measured with a 532 nm laser (A), and a 785 nm laser (B), along with that of the FRET polymers with the same lasers (C and D, respectively). The spectra for the PS-FRET microfibers showed no difference to that of the pure PS microfibers (data not shown).

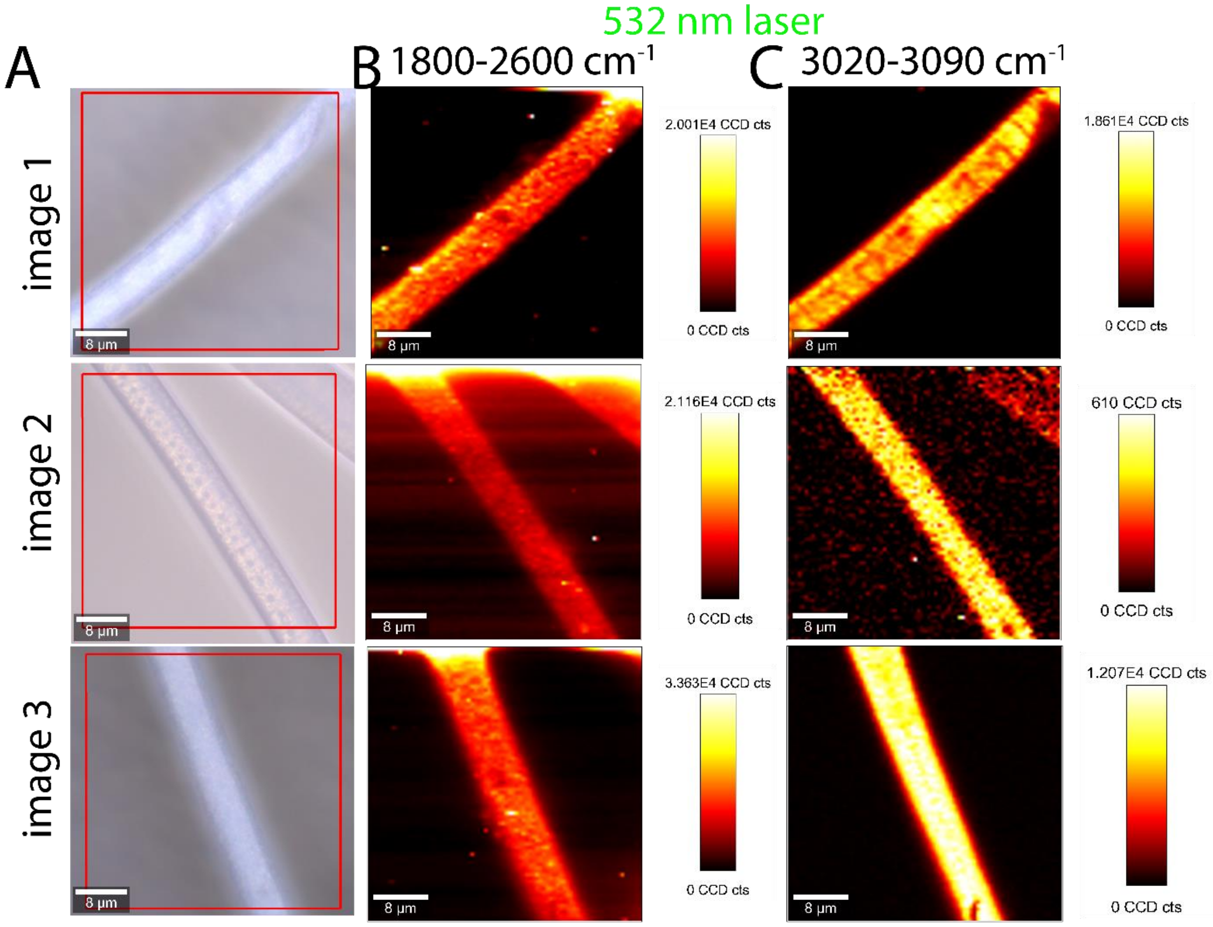

Figure S6. Optical images of the PS-FRET fibers (A), with corresponding Raman mapping over 1800-2600 cm^{-1} (B) and 3020-3090 cm^{-1} (C), which corresponds to fluorescence and PS signals, respectively, under the 532 nm laser (Figure S5).

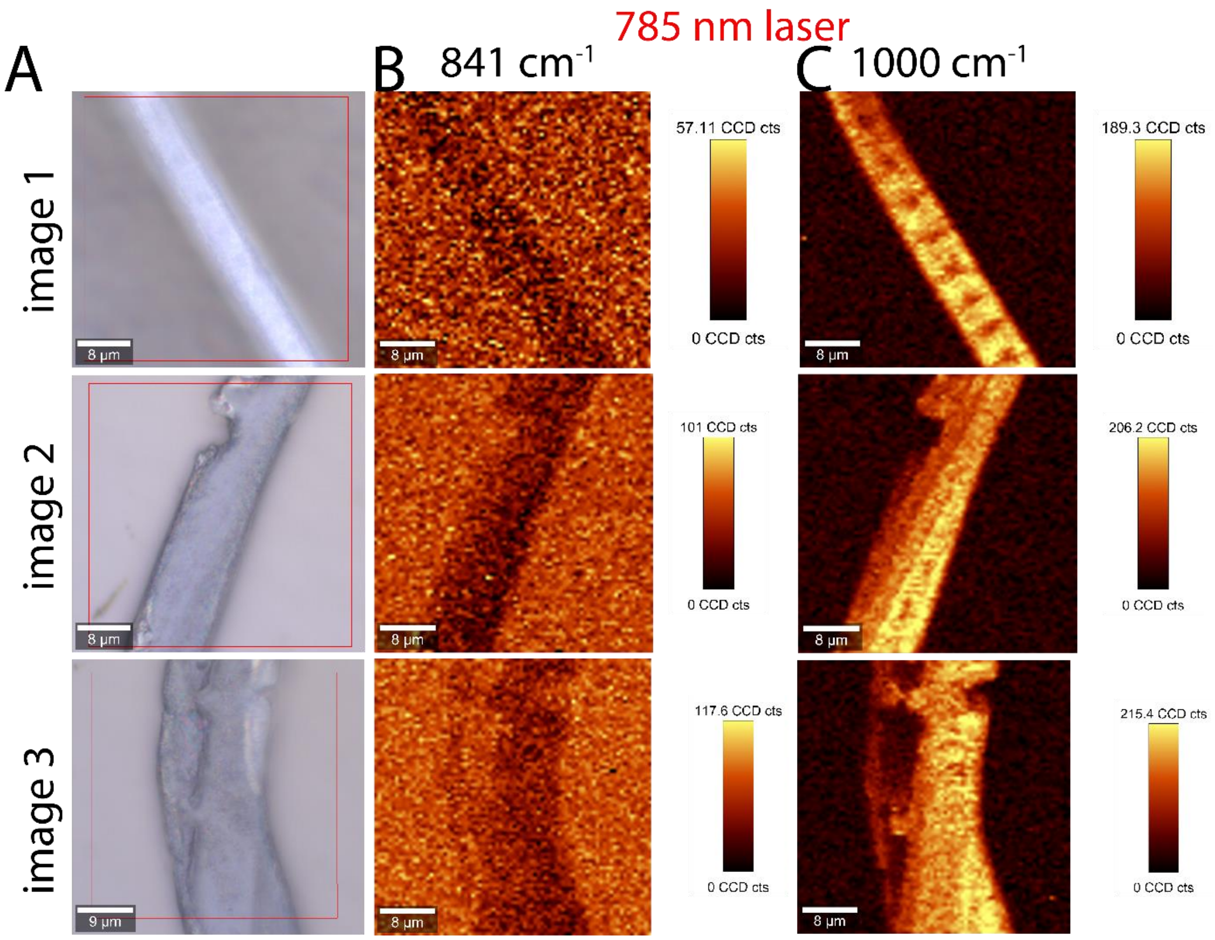

Figure S7. Optical images of the PS-FRET fibers (A), with corresponding Raman mapping at 841 cm^{-1} (B) and 1000 cm^{-1} (C), which corresponds to specific fluorescence and PS signals, respectively, under the 785 nm laser (Figure S5). We note that the fibers in column B correspond to the unique peak in the NBD spectrum (Figure S5), and the lack of clear signal in the fibers indicates that the concentration of the FRET polymer is too low to produce an NBD signal. The dark region of the fiber is most likely a shadow artifact of the fiber itself, with minimal differences from the background.

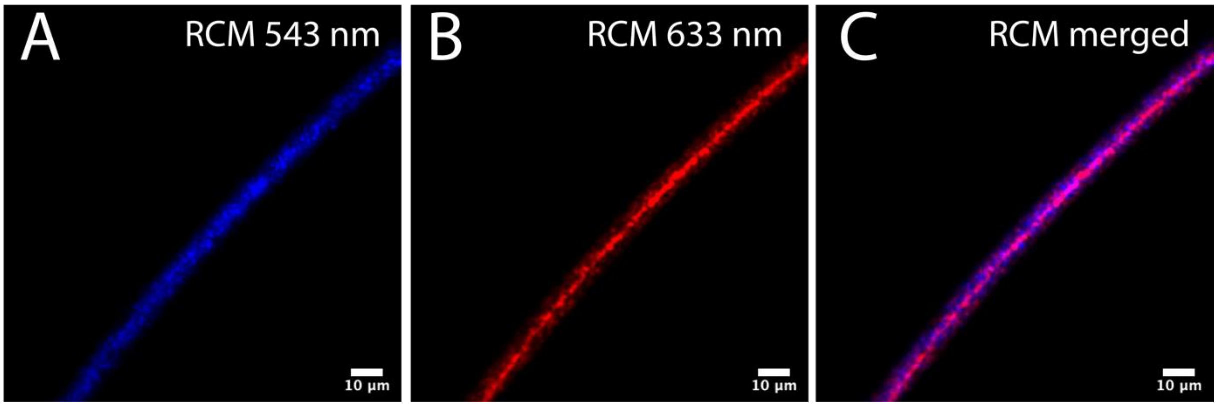

Figure S8. RCM comparison of a PS microfiber at two wavelengths of A) 543 nm, B) 633 nm, and C) a composite of the two. Scale bars indicate 10 μm.

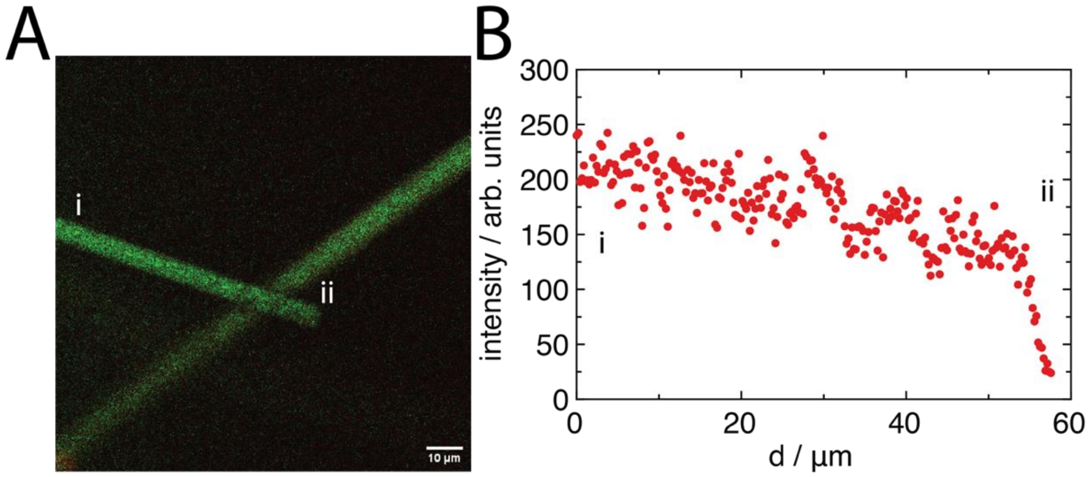

Figure S9. A) CLSM image of a fiber end (merged donor and acceptor channels), along with a line profile (between regions i and ii) of the intensity. Scale bar indicates 10 μm.

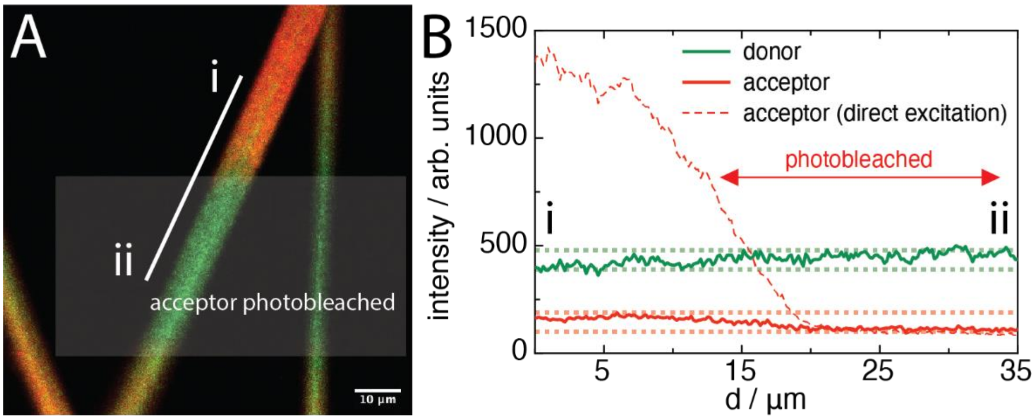

Figure S10. A) CLSM combined image of donor and direct acceptor excitation of microfibers where in the grey boxed region the acceptor was photobleached, along with B) line profile analysis along this same approximate region. The horizontal dotted green and red lines are to guide the eye as to the increase in donor intensity with a decrease in acceptor intensity. Scale bar indicates 10 μm.

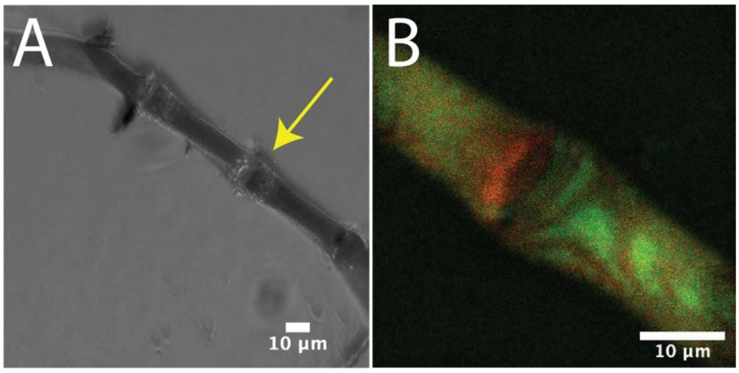

Figure S11. A) WF image of a section of damaged fiber (via micromanipulator) along with B) a CLSM merged image corresponding to the region of interest (yellow arrow in A). Note that this fiber was not broken, only dinted. Scale bars indicate 10 µm.

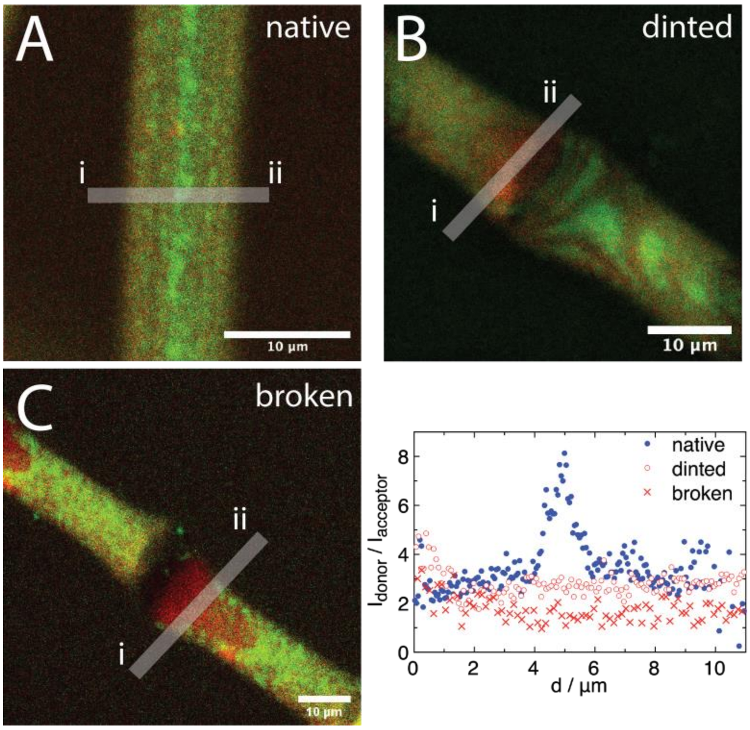

Figure S12. CLSM images of a A) native, B) dinted, and C) broken microfiber, along with line profile analysis across the perpendicular axis. The light grey lines in A-C indicate the approximate regions over which the line profiles were calculated. Scale bars indicate 10 µm.

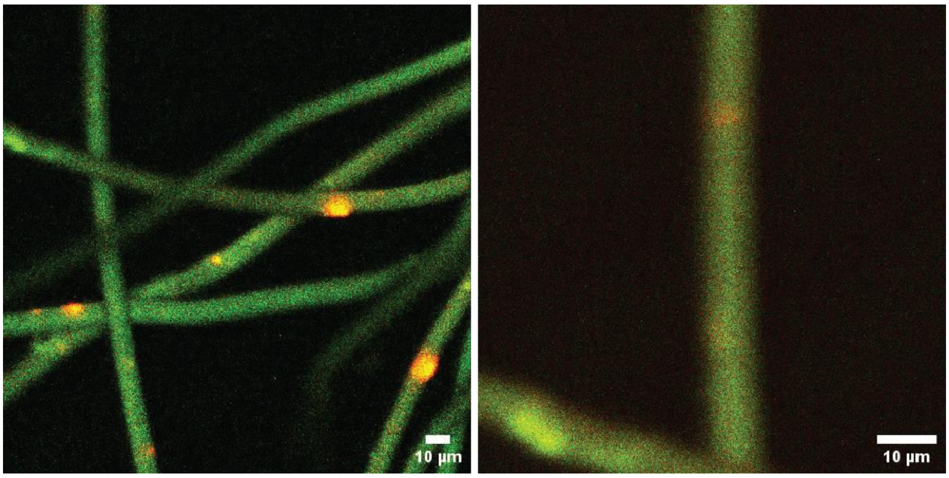

Figure S13. CLSM images of the PS/FRET fibers taken 10 months after the electrospinning processes to check the stability of the system. Note the persistence of the spine-like structure (zoomed image, right side). Scale bars indicate 10 μm.
